# Airbag-related chest wall burn as a marker of underlying injury: a case report

**DOI:** 10.1186/1752-1947-2-91

**Published:** 2008-03-24

**Authors:** Simon J Monkhouse, Michael D Kelly

**Affiliations:** 1 Department of Surgery, Frenchay Hospital, Bristol, UK

## Abstract

**Introduction:**

This case of a man who sustained an airbag-induced thoracic injury and burn, highlights the potential harm that can be caused by airbags. It also serves to illustrate that a surface burn which looks small and benign can actually be a surface marker of a more serious injury. Staff working in emergency departments need to be aware of the risk of possible airbag-associated injuries.

**Case presentation:**

A 65-year-old man was the driver in a frontal collision. He was wearing a seatbelt. The airbag was activated and caused a superficial chest wall burn. Initial chest x-rays were unremarkable but following deterioration in his condition, a computed tomography scan revealed a serious sternal fracture. The location of the fracture was marked on the surface by the burn.

**Conclusion:**

Airbags can cause significant chest wall injuries and burns. Surface burns at the point of impact should not be dismissed as trivial as the forces involved can cause significant injury. We recommend that all people with chest wall injuries and/or burns due to airbags should have more detailed chest imaging as initial emergency radiographs can be falsely reassuring.

## Introduction

It has been well documented in the literature that the introduction of frontal airbags has had a significant impact in reducing mortality and serious injury from motor vehicle accidents [1]. However, the mechanism of action and speed of deployment of airbags can be associated with injury and morbidity. Injuries to the eyes and ears are particularly well reported and there is evidence that the incidence of lower limb injuries has increased as a result of redistribution of the forces associated with a crash [2]. Of particular interest in this case is the increased risk of chest wall injury and burns.

A prospective European study [3] looked at 188 motor vehicle accidents and analysed the relative risk of victims sustaining significant chest trauma in the presence or absence of an airbag. The conclusion was that sustaining such an injury was highest in drivers wearing a seatbelt in a car with a frontal airbag. This is an interesting finding which challenges the safety record associated with airbags. The distance the driver is from the airbag is also significant. Airbag manufacturers tend to recommend a minimum distance of 25 cm between the driver and the steering wheel to maximise the beneficial effects of the airbag and to minimise adverse effects. If the driver is too close, then the expansion of the bag is impeded by the patient's torso and this may cause shearing forces that can lead to injury. The expanding bag is forced upwards as a consequence which can cause facial and neck injuries.

The incidence of burns following airbag deployment has been noted at 1.53% [4]. A whole array of burns has been recorded, mainly ocular and facial. A rapid deceleration of the vehicle triggers the combustion of various chemicals, including sodium azide, sulphur and potassium nitrate resulting in rapid nitrogen production which inflates the bag [5]. Bag rupture results in exposure to hot metal combustion pipes and spillage of alkaline chemicals.

In this case, the driver suffered burns and chest wall trauma from airbag deployment. The occult presentation and significance of the injuries is outlined.

## Case presentation

**Figure 1:**
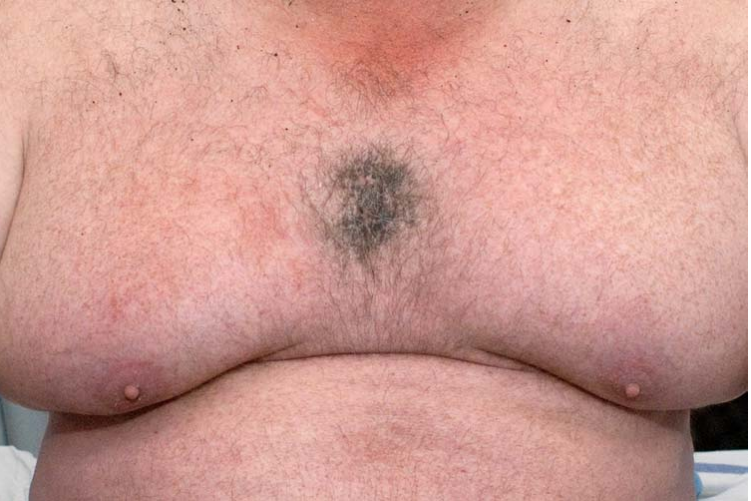
Chest wall burn caused by airbag deployment.

**Figure 2:**
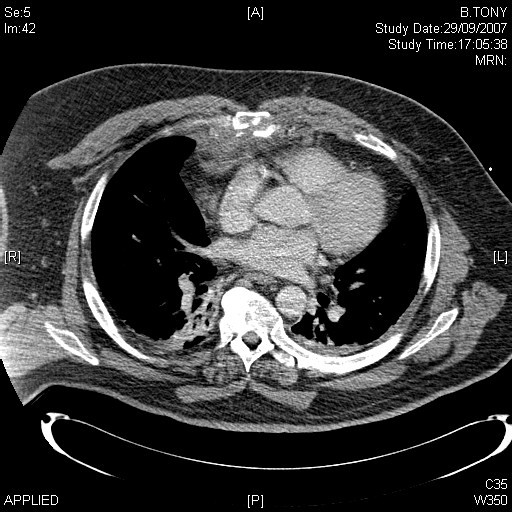
CT scan showing a comminuted fracture of the sternum and retrosternal haematoma.

A 65-year-old man was involved in a moderate speed, frontal impact road traffic accident. He was restrained by a three-point fixation seat belt and his frontal airbag deployed appropriately in the collision. He was assessed by paramedics at the scene and was brought to the emergency department for assessment, although it was felt he had no serious injuries apart from a forehead laceration. His head was sutured and he was assessed as fit for discharge. However, he reported increasing chest pain, which was unresponsive to simple analgesia. A routine chest x-ray was performed and reported as showing no evidence of pneumothorax or rib injury. He was admitted to hospital overnight. The following morning, he was reporting chest pain and was short of breath. Examination revealed a small, distinct chest wall burn (Fig. 1) and a CT scan of the thorax demonstrated comminuted sternal and manubrium fractures (Fig. 2). There was also a significant retrosternal haematoma. Electrocardiogram and echocardiography were unremarkable. He was admitted for conservative management which included serial observation, chest physiotherapy and opiate analgesia. He made a steady recovery and was discharged from hospital four days later.

## Conclusion

This case is of enormous importance to emergency staff. The burn was exactly over the point of sternal fracture and served as a marker of serious underlying injury. The highly localised nature of the burn indicates that the forces involved in the airbag deployment were focused on this point. The educational point here is that in a patient with a chest wall burn sustained during airbag deployment, treating clinicians should be suspicious of an underlying thoracic injury. Appropriate imaging is necessary to rule out a serious chest wall injury as plain films and clinical examination can be misleading. In addition, all drivers should be encouraged to sit back from the steering wheel at a distance of at least 25 cm to minimise the risk of injury due to adverse explosive forces.

## Competing interests

The author(s) declare that they have no competing interests.

## Authors' contributions

SM wrote the case report and performed the literature search. MK edited the manuscript and organised the medical photography. Both authors read and approved the final manuscript.

## Consent

Written informed consent was obtained from the patient for publication of this case report and accompanying images. A copy of the written consent is available for review by the Editor-in-Chief of this journal.
